# Supplementary material for: Plotter Cut Stencil Masks for the Deposition of Organic and Inorganic Materials and a New Rapid, Cost Effective Technique for Antimicrobial Evaluations
Source: Micromachines (Basel). 2022 Dec 21;14(1):14. doi: 10.3390/mi14010014 (PMC9864392; doi:10.3390/mi14010014)
Supplement: Supplementary file 1 [file micromachines-14-00014-s001.zip › micromachines-2070386-supplementary.pdf]

Supplementary

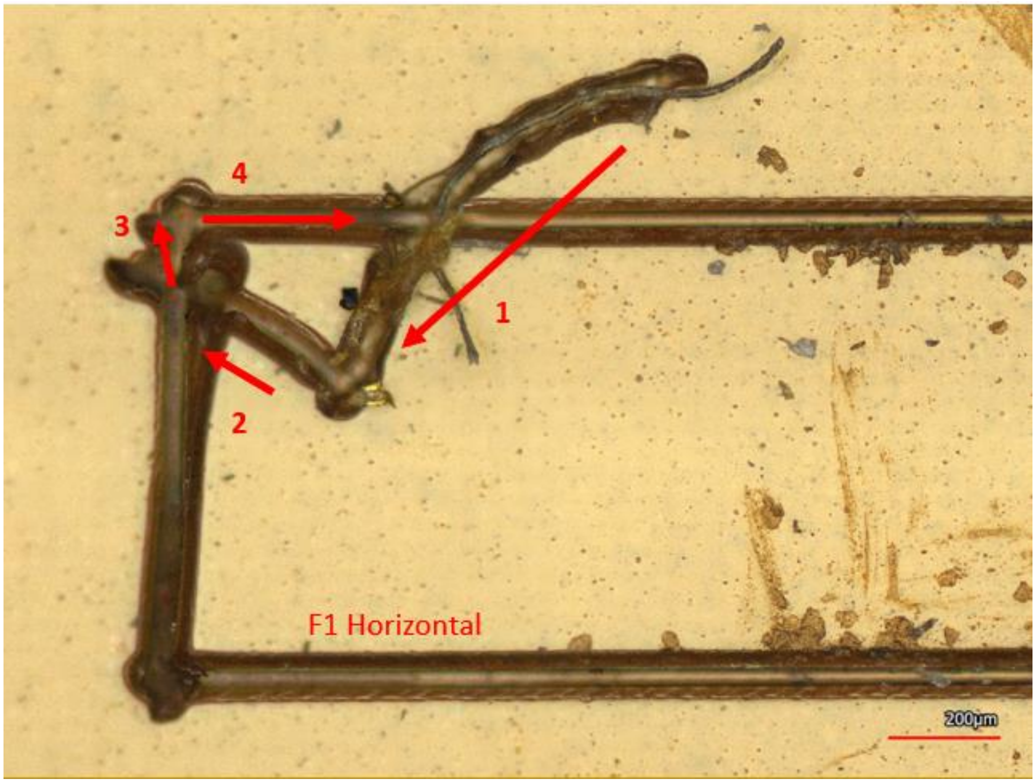

Fig. S1. Drag Knife Method: Steps for turning rotations and directions of knife movement shown with red arrows.

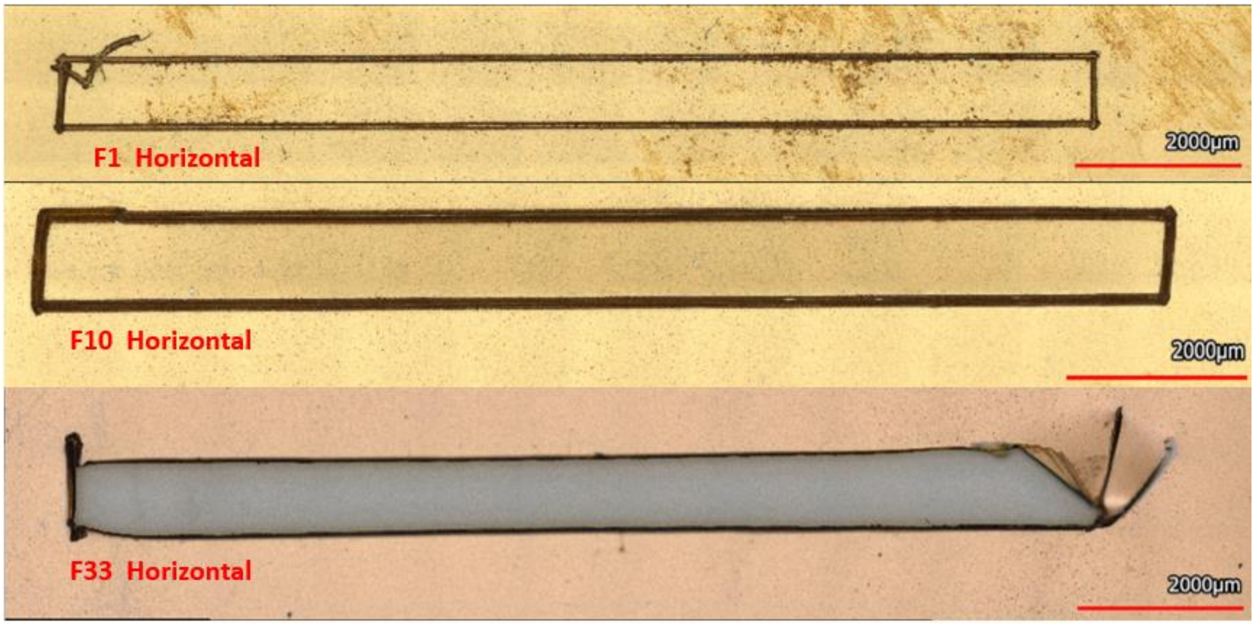

Fig. S2. Forces 1, 10 and 33 showing cutting parameters that do not cut through the Kapton® or cause excessive tearing near the edges of the cut channel.

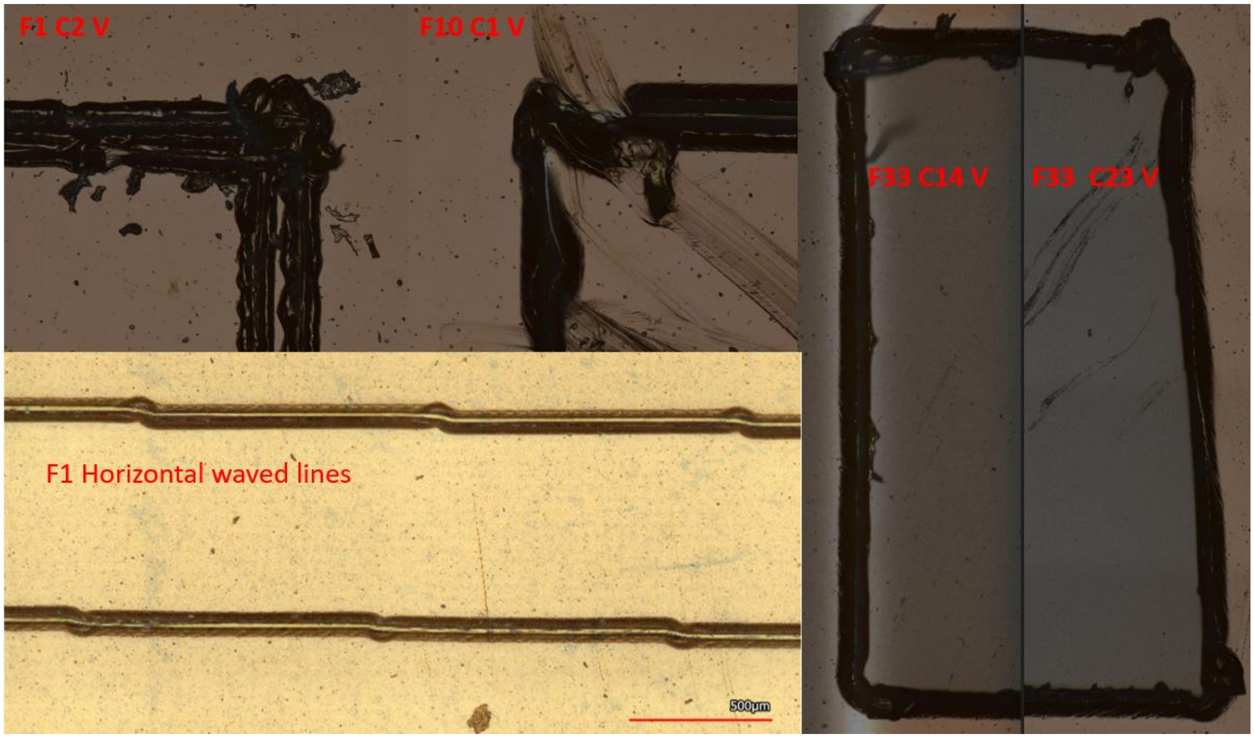

Fig. S3. 3D confocal images of "Bad Cuts". These consist Vertical and Horizontal cuts showing Force 1 Corner 2, Force 10 Corner 1, Force 1 Horizontal cut and Force 33 showing over extension on the corners

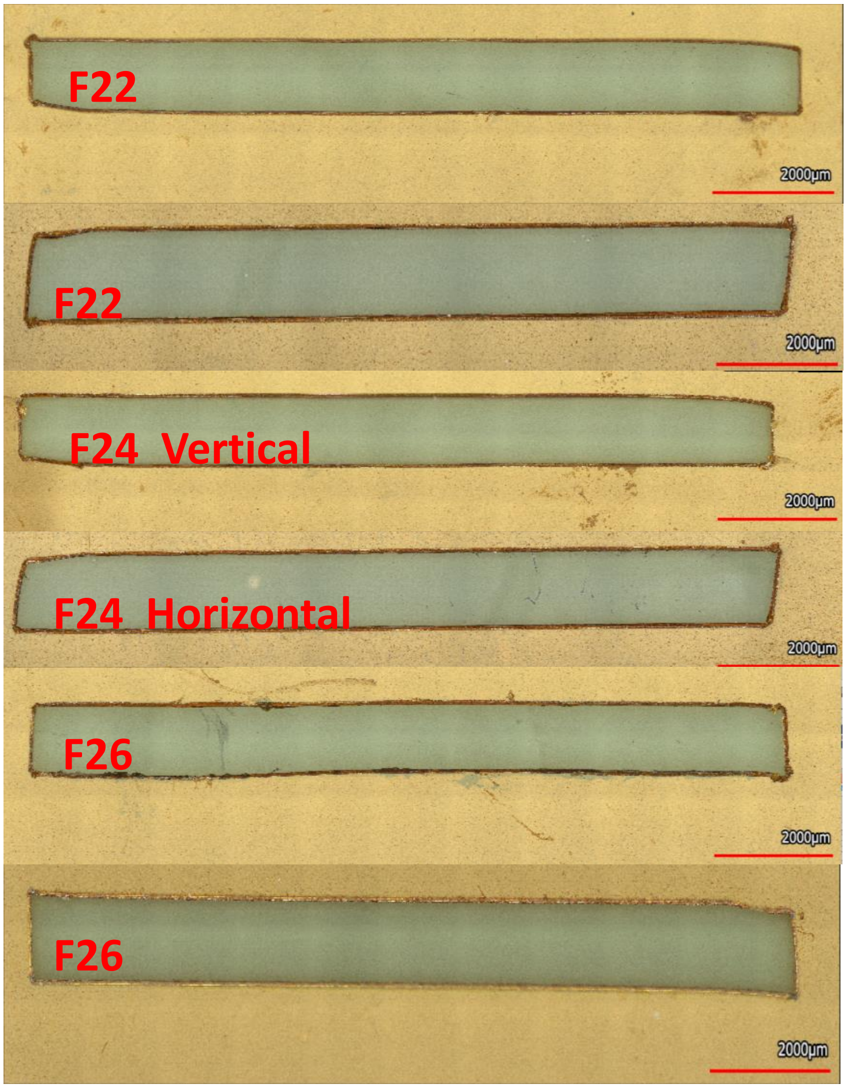

Angle of Corners

93.72 ± 1.02

92.48 ± 2.855

95.44 ± 0.97

93.32 ± 1.98

94.18 ± 1.31

91.18 ± 1.31

Fig. S4. Image of Horizontal and Vertical cuts, validated to be "Good Cuts" with the Average Angles of the corners and Standard Deviations.

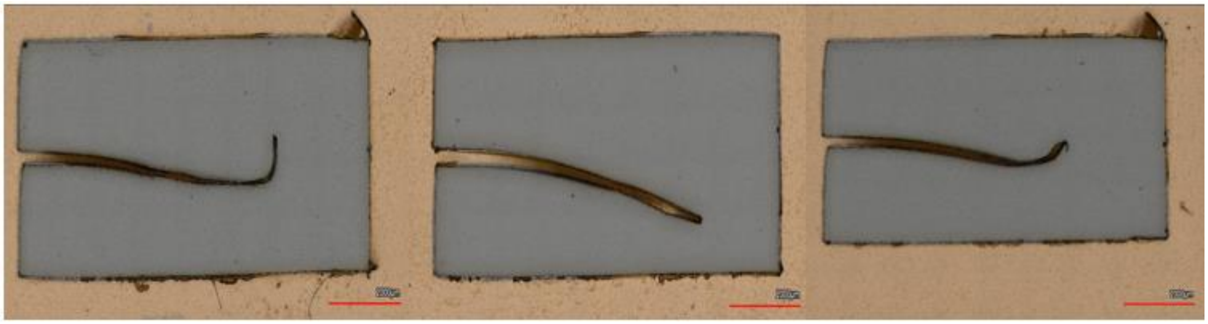

Fig. S5: Optical image of Square Channels below 300 μm with plastic deformation.

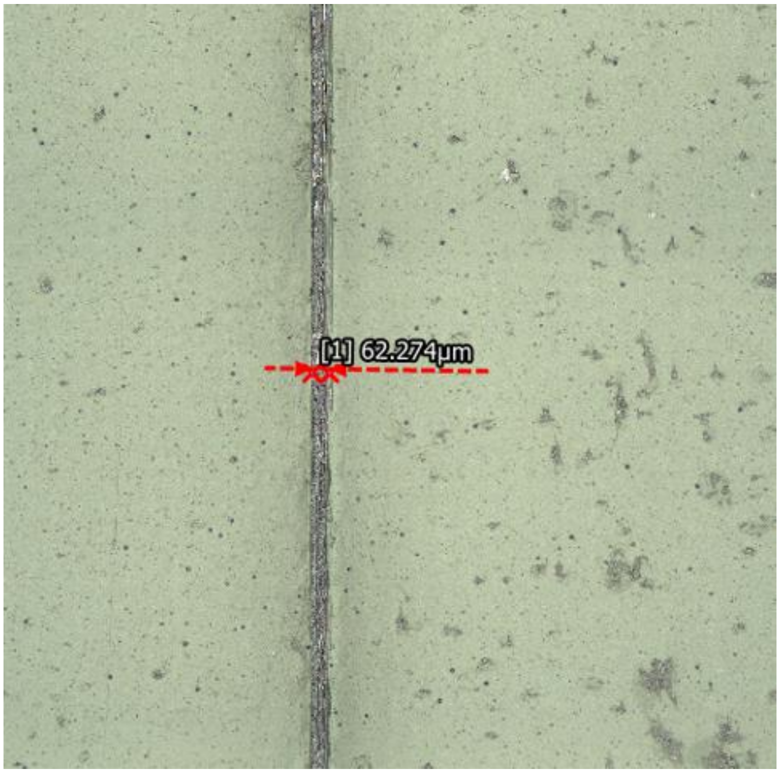

Fig. S6. Optical image of a single line with a width of ~62.3 μm. This feature size could not be replicated, however it represents for the first time demonstration of feature sizes below 100 μm using plotter cutters.

Table S1. Plotter cutters with applications, resolutions, advantages, and disadvantages.

Plotter Cutter	Application Investigated	Resolution Pros and Cons
[12] Graphtec6000-40 ($2000)	Features were cut into a pressure sensitive double-sided adhesive (polyester) in straight, serpentine, square and zig zag channels for microfluidics.	Channel resolution widths below 200 μm were not achieved in this work. With the 30° blade 208.07 ± 9.09 μm, with the 45° blade 231.84 ± 26.5 μm were achieved. Designs can be developed in minutes with spacings between designs being 1.5 mm. The roughness of the channels is high.
[17] FC5100A-75 Graphtec ($4000)	Features were cut in both straight and serpentine channels in order to see the minimum resolution in a variety of materials, to create shadow mask for electroplating and molds.	The smallest straight positive and negative channel resolutions were 32 μm and 15 μm respectively. The cutter also reached 78 ± 23 μm in serpentine channels. Deviations were less than 10 μm from drawn dimensions, with large discrepancies in thick films.
[18] Quickutz ® Silhouette ($300)	Microfluidic devices were cut into double sided pressure sensitive tape 50 μm thick.	The report stated that the resolution reached 200 μm, however did not discuss actual cut dimensions only showed that the cuts did not fail.
[19] Silhouette Cameo™ ($150)	Straight channel pressure driven microfluidic devices were cut into omniphobic R^F paper.	Resolutions reached 45 μm. The treated material was a hydrophobic reactive substrate. The devices made required pressure to begin flow, and the gas permeable substrate led to partial evaporation of the liquid.

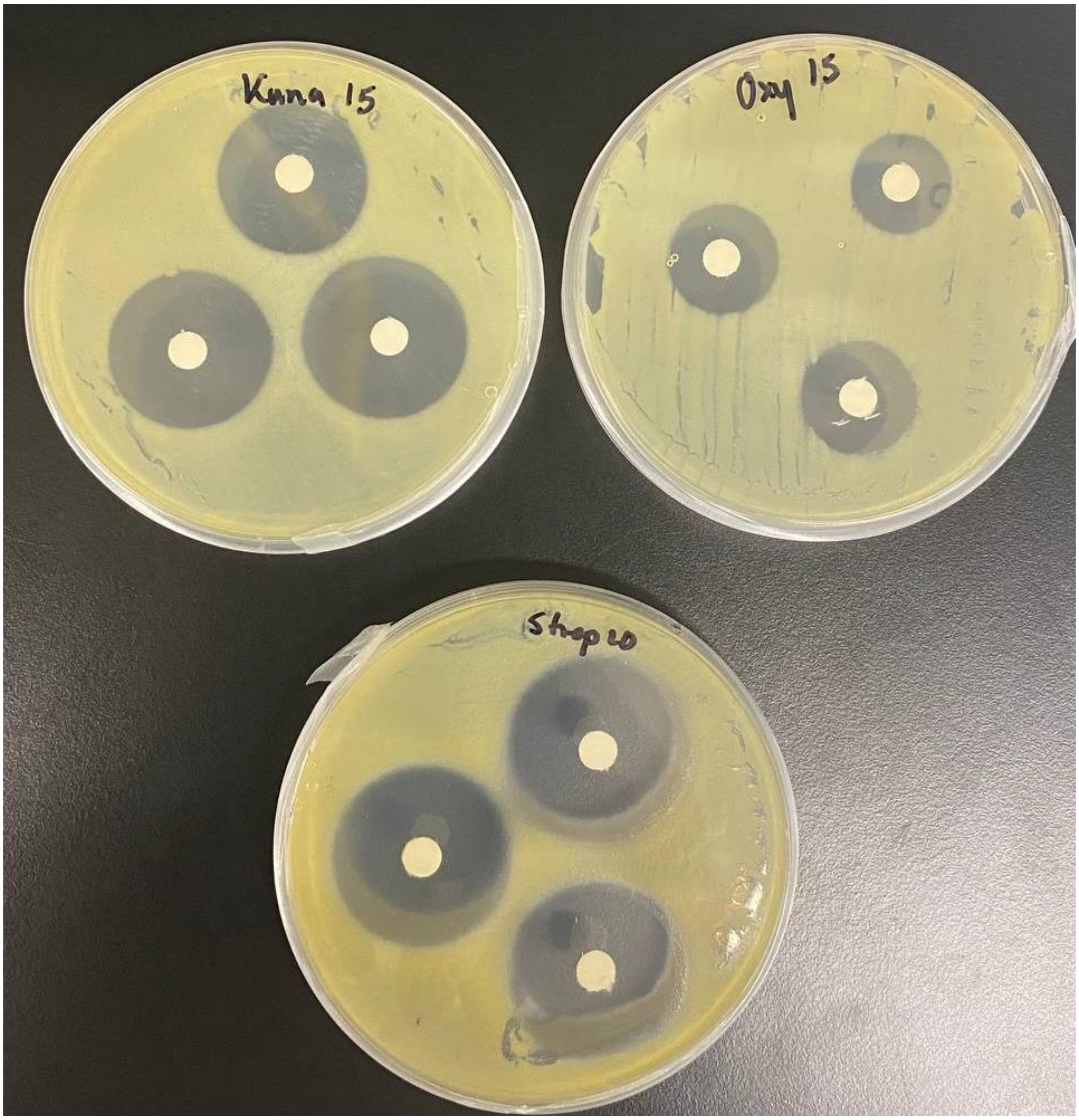

Fig. S7. Example Kirby Bauer Disk Diffusion Tests (DDTs) for Streptomycin 20 μg, Oxytetracycline 15 μg and Kanamycin 15 μg.

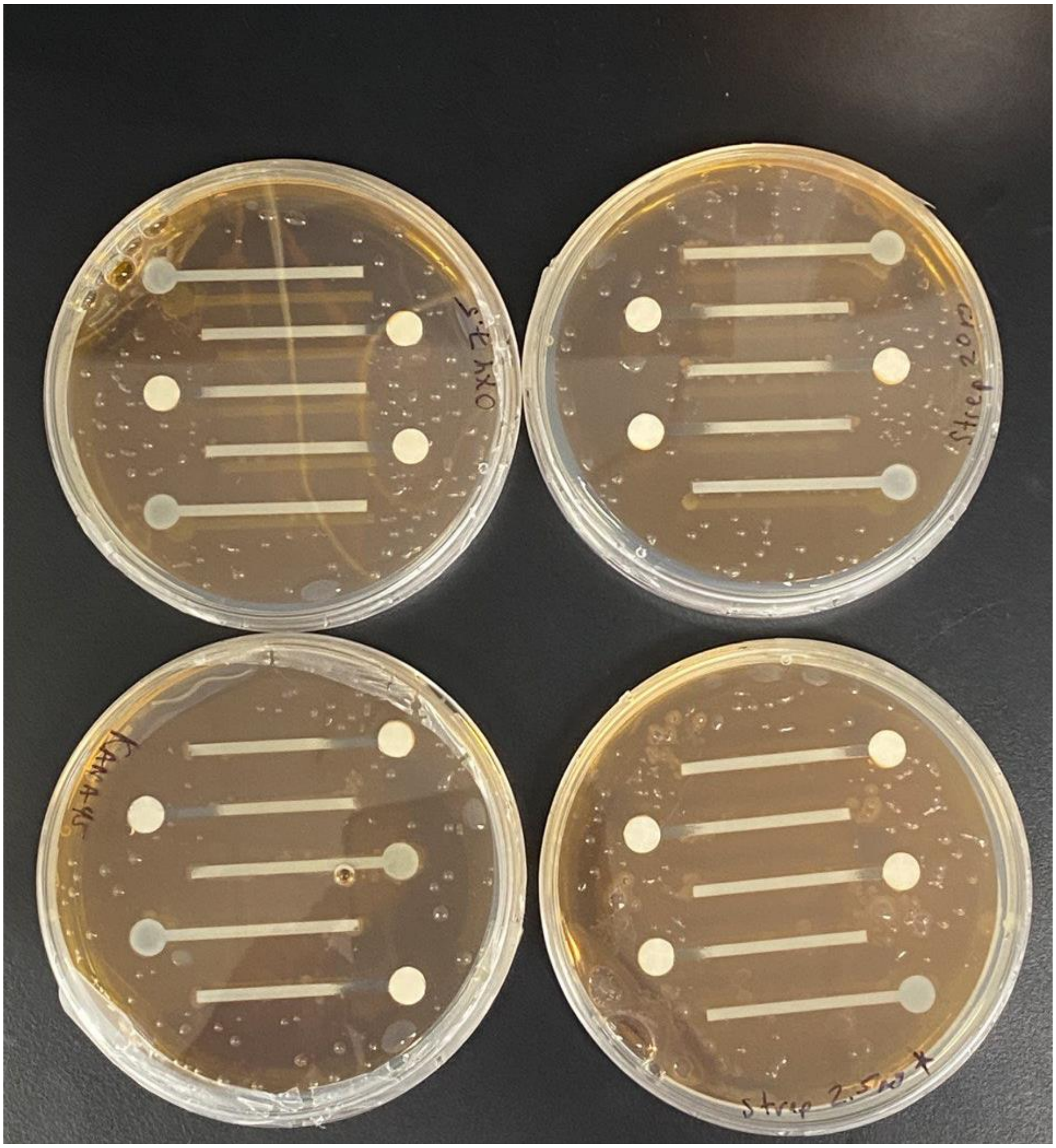

Fig. S8. Example images of SDDT for Kanamycin 45 μg, Streptomycin 2.5 μg, Oxytetracycline 7.5 μg and Streptomycin 20 μg.
